# The Role of Chlamydia Trachomatis in the Pathogenesis of Cervical Cancer

**DOI:** 10.7759/cureus.21331

**Published:** 2022-01-17

**Authors:** Ana P Arcia Franchini, Beshoy Iskander, Fatima Anwer, Federico Oliveri, Kakargias Fotios, Priyanka Panday, Pousette Hamid

**Affiliations:** 1 Research, California Institute of Behavioral Neurosciences & Psychology, Fairfield, USA; 2 Internal Medicine, Bon Secours Mercy Health - St. Elizabeth Youngstown Hospital (NEOMED), Ohio, USA

**Keywords:** human papillomavirus, chlamydia trachomatis, coinfection, cervical intraepithelial neoplasm, cervical cancer

## Abstract

Chlamydia trachomatis (CT) is an obligate intracellular, Gram-negative bacterium that causes a variety of infections in both humans and animals. It is the causative agent of one of the most common sexually transmitted infections (STIs) with sequelae such as pelvic inflammatory disease, ectopic pregnancy, and infertility. Furthermore, Chlamydia infections have been epidemiologically linked to cervical cancer (CC) in patients with human papillomavirus (HPV) coinfection. However, a molecular mechanism linking Chlamydia to CC is yet to be established, and we still do not know if more aggressive diagnosis and treatment of Chlamydia could possibly lead to lower incidences of CC and associated mortality. Since CC is a leading cause of death among women worldwide, and HPV infection alone is insufficient to cause cancer, our goal was to determine the link between Chlamydia, HPV, and CC. This literature review aimed to understand the pathologic model of CC and how Chlamydia might induce or promote carcinogenesis alone or alongside HPV. In addition, we compared multiple studies attempting to associate Chlamydial infection with CC in different populations and aimed to determine whether there is an epidemiological correlation or not.

## Introduction and background

Cervical cancer (CC) is the fourth most common cancer among women worldwide, with 604,127 new cases documented in 2020. It is also the fourth leading cause of cancer-related death in the female population after breast, lung, and colorectal cancers, with 341,834 deaths reported [1]. Many risk factors have been associated with CC, most notably human papillomavirus (HPV). HPV is a non-enveloped double-stranded DNA virus that can be classified into cutaneous or mucosal and can be either high-risk (hrHPV) or low-risk (lrHPV). Even though there are over 200 HPV genotypes, common lrHPV 6, 11, 42, 43, and 44 more often cause benign cutaneous lesions in the form of warts and only very rarely develop into tumors; by contrast, hrHPV 16, 18, 31, 33, 34, 35, 39, 45, 51, 52, 56, 58, 59, 66, 68, and 70 are associated with cervical neoplasia, 70% being caused by HPV 16 and 18 [2].

HPV has often been described as a necessary but insufficient cause of CC, in that even though HPV DNA can be detected in around 99% of CC cases, the majority of HPV infections are asymptomatic and clear spontaneously within one to two years. Only a small number of exposed women will present with persistent infection and progress to cervical changes [3-5]. Therefore, many studies have been conducted in order to investigate other risk factors that could be involved in the pathogenesis of CC, either by enhancing susceptibility or facilitating the persistence of HPV infection [6]. The additional discovered risk factors include HIV, immunosuppression, multiple sexual partners, age of first coitus, cigarette smoking, multiparity, oral contraceptives, low socioeconomic status, nutritional deficiencies, and the presence of other sexually transmitted infections (STI) such as Chlamydia trachomatis (CT) [3,5,7].

Sexually transmitted diseases are a common healthcare challenge in communities worldwide, with one million STIs estimated to be acquired every day around the world. It is estimated that there are 357 million new infections due to one of these four common STIs each year, including Chlamydia, gonorrhea, syphilis, and trichomoniasis [1]. Among the previously mentioned, Chlamydia is the most common bacterial STI, representing 20-40% of all STIs with more than 1.4 million infections in the United States alone [7]. It is important to point out that the number of CT infections may actually be more than reported since around 80% of CT infections may be asymptomatic, establishing persistent infections by several mechanisms including antibiotic resistance, immune evasion, and apoptosis [3,8].

CT is a Gram-negative, obligatory intracellular bacterium with a small genome that is capable of causing several diseases in humans and animals [9,10]. It can be categorized into at least 19 serotypes, including serovars A to C, which relate to trachoma; D to K, which relate to urogenital infections and pelvic inflammatory disease leading to scarring, ectopic pregnancies, and infertility; and L1 to L3, which are associated with lymphogranuloma venereum [8,11]. As an intracellular bacterium, CT not only leads to epithelial disruptions and micro-abrasions, but it can also cause great alterations to gene expression and protein production at the transcriptional, translational, and post-translational levels [3,7,12]. Consequently, CT can induce local secretions of immune mediators, stimulating the production of reactive oxygen species and free radicals. This, in turn, promotes persistent damage of mucosal barriers and cell-mediated immunity and accentuates MMP-9 expression contributing to tumor progression [8,13]. It has also been previously evidenced in cultured cells that CT infection leads to centrosome amplification, spindle defects, and chromosomal instability, which eventually leads to cell transformation [10]. We hypothesize that this induction to cervical metaplasia by CT is what creates target cells for HPV coinfection and its interference with the immune response is the reason for persistent infection [14]. 

Despite many epidemiological studies contributing to the growing collection of data asserting the role of CT in CC development, many others have reported no correlation between the two [3,9,10,15]. Even though both HPV and CT share common transmission routes and risk factors, there is a lack of physiologically relevant infection models that could clarify the mechanisms of the infection’s progression and the development of cancer [8,12]. There is also little evidence regarding the benefits of continuous screening and treatment of CT to reduce the incidence of CC. That said, a direct correlation between CT and CC in women with or without HPV coinfection is still unclear. The objective of this literature review is to gain an understanding of the existing research regarding the pathophysiological role of CT in the progression of CC and thereby clarify if constant screening and proper treatment of chronic CT could lower the number of new cases of CC and the consequent deaths attributed to it.

## Review

We employed a search strategy using electronic databases PubMed and Google Scholar, covering the period between the years 2011 to 2021. The following keywords ‘Chlamydia trachomatis’; ‘cervical cancer’; ‘cervical intraepithelial neoplasia’; ‘Human papilloma virus’; and ‘coinfection’ yielded a large number of articles by themselves, and hence they were combined to narrow down our search. The MeSH headings ‘Chlamydia trachomatis’; ‘Uterine cervical neoplasms’; and ‘Papillomavirus infection’ were also included and were narrowed down by combining them with relevant subheadings.

The set of articles elicited by each search strategy was then filtered according to relevance and availability, choosing only those with free full texts and abstracts in the English language that were applicable to our review. Those selected include meta-analysis, controlled trials, observational studies, review articles, and animal studies. After the records were assessed and ineligible articles ruled out, a total of 30 remaining articles were selected to serve as evidence for our literature review.

**Figure 1:**
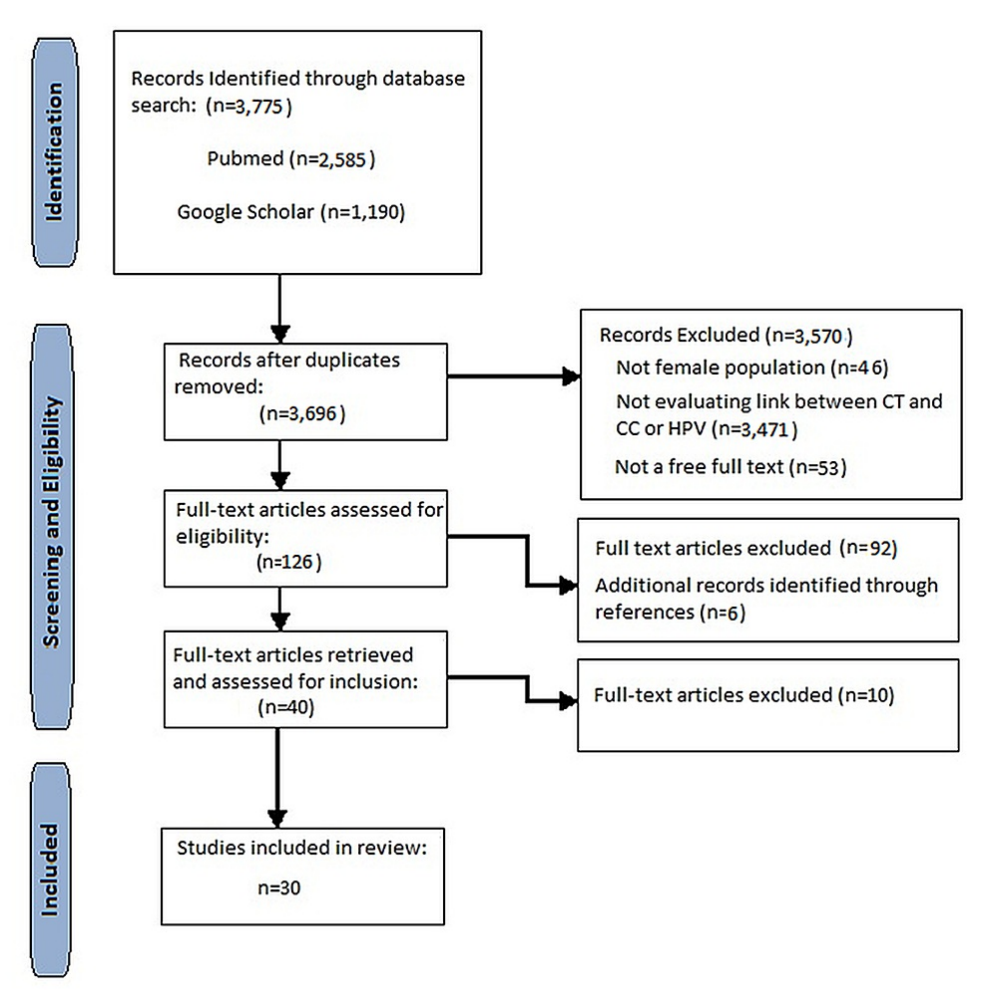
Flow diagram of the literature search CT: Chlamydia trachomatis; CC: cervical cancer; HPV: human papillomavirus

Figure 1 depicts the flow diagram of the literature search on PubMed and Google Scholar up until April 16, 2021.

Discussion

Cervical Intraepithelial Neoplasia and Carcinogenesis

Carcinogenesis is the process by which normal, healthy cells transform into cancer cells. The early events leading to cervical epithelial transformation are attributed to the integration of the HPV genome into the host’s chromosomes [16]. With time, the HPV-infected cells will accumulate DNA alterations and enable a series of epigenetic events that will allow its continuous viral replication, setting the perfect stage for neoplastic changes [2,17]. This can only be possible with persistent infection and the infiltration of the virus into the epithelial cells at the basal layer where viral particles are released to assist with the integration of its genome into the host’s [2].

**Figure 2:**
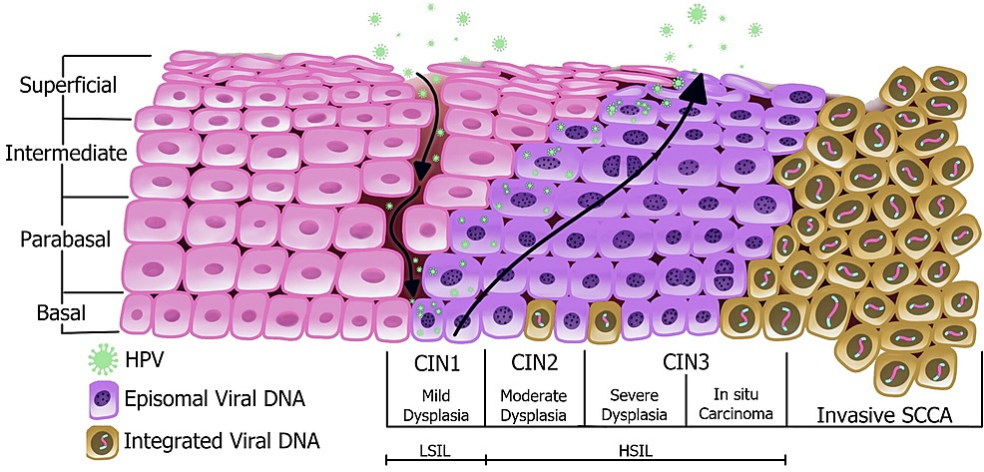
Pathogenesis of HPV in cervical cancer HPV: human papillomavirus; CIN: cervical intraepithelial neoplasia; LSIL: low-grade squamous intraepithelial lesion; HSIL: high-grade squamous intraepithelial lesion; SCCA: squamous cell carcinoma [19]

Figure 2 illustrates the infiltration of HPV into the basal layer through lesions and microabrasions, and its subsequent integration and replication, taking over the cervical epithelium in stages with the help of its oncoproteins [16-18]. When the lower one-third of the epithelium shows dysplastic changes, it is classified as cervical intraepithelial neoplasia (CIN) 1, also known as the low-grade squamous intraepithelial lesion (LSIL). When two-thirds of the epithelium is dysplastic, it is known as CIN 2, and CIN 3 occurs when more than two-thirds are affected. CIN 2 and CIN 3 are both classified as high-grade squamous intraepithelial lesions (HSIL) [16].

HSIL can develop within three to five years after the initial infection, and the progression towards invasive cancer can take two to three decades more; but in order for this to occur, the virus must first evade the host’s immune systems [2,17]. It has been described that HrHPV can inhibit major histocompatibility complex (MHC) class 1 via its viral protein E5, and E6 is capable of inhibiting toll-like receptors (TLR) expressed by natural killer T cells. Another study has demonstrated that the expression of the TLR-9 gene differs throughout the stages of CIN, being at its lowest during CIN 1 and at its highest in samples with squamous cell carcinoma. Nonetheless, the ongoing expression of E6 and E7 oncoproteins and their downregulation of TLR surely interfere with interferon response, perpetuating its evasion of the host’s immune system [2]. There is also evidence of decreased production of proinflammatory cytokines, and a subsequent decrease in immune cell attraction. This is accomplished by HPV’s suppression of NF-κB by increasing the expression of interferon-related developmental regulator 1 (IFRD1) through the upregulation of epidermal growth factor receptors (EGFR) [18].

In addition, a comparison study between healthy controls and patients with CIN or uterine CC concluded that the latter presented a lower proportion of Th1 cells and a higher one of Th2, Th17, and regulatory T cells (Tregs). Other findings were increased concentrations of IL-4, IL-10, IL-17, IL-28, and TGF-βI, and decreased concentrations of INF-γ, suggesting that HPV-triggered immunologic dissonance maintains the infection’s continuation [20].

Nonetheless, the majority of hrHPV infections are controlled by the immune system and infection alone is not enough to trigger the changes necessary for the progression to CC [2]. There are significant genomic alterations that must take place for precancerous lesions to become cancerous [2,18]. Once HPV has evaded the immune system and integrated into the cell, E6 and E7 oncoproteins interrupt the cell cycle’s checkpoints. This is achieved by the inhibition of cyclin-dependent kinase (CDK) inhibitors p21, p27, and p16, and the degradation of p53 and retinoblastoma (pRB) [2]. The effect of E6 oncoprotein on p53 allows the continuation of viral genome replication in conjunction with cellular DNA, bypassing any mechanisms of repair by the host cell [2,17]. The now-stable production of episomal HPV is needed for the integration of HPV DNA into the host genome. The degradation of pRB caused by E7 oncoprotein will cause the cell to enter the S phase of the cell cycle promoting cell proliferation [2]. As the resulting daughter cells differentiate, there is upregulation of viral differentiation-dependent promoters, resulting in increased expression of viral genes, including E6 and E7, perpetuating viral genome replication [17].

When the HPV-infected cells reach the upper layer of the cervical epithelium, the virus reaches the final stage of its life cycle, resulting in the formation of new viral particles that are shed from the terminally differentiated cells ready to infect healthy basal cells again [17]. As the infection continues and genes in charge of cell cycle regulation and metastasis are over-expressed, the stage of invasive carcinoma begins. Many studies report that from CIN 2 and 3 to CC, there is high cellular stress due to overcrowding of cells. The invading cells now overcoming the barriers set by the epithelial cells and basement membrane need more nutrients and activate genes that will trigger angiogenesis, epithelial cell differentiation, extracellular matrix (ECM) organization, and collagen fibril organization (Table 1) [2].

**Table 1:** Non-comprehensive list of genes involved in cervical cancer progression

Genes identified	
Phosphoinositide-3-kinase (PIK3CA)	Alpha actinins (ACTN1)
Vascular endothelial growth factor A (VEGFA)	Fibronectin 1 (FN1)
Integrin subunit alpha 1 (ITGA1)	Collagen type 1 (COL1A1)
Protein tyrosine kinase (PTK2)	Collagen type 2 (COL1A2)
Integrin subunit beta 1 (ITGB1)	Syndecan 2 (SDC2)

HrHPV types 16 and 18 are associated with at least 90% of all cervical carcinomas, but only a small percentage of women exposed to HPV will progress to have CC. Therefore, there must be other risk factors in conjunction with HPV in the development of CC, and these proposed cofactors include cigarette smoking, early sexual activity, low socioeconomic status, multiparity, oral contraceptives, chronic inflammation, nutritional deficiencies, HLA status, gene polymorphisms, host genetic and immunologic responses, and coinfection with other microorganisms such as herpes simplex and CT [3-5,21].

Carcinogenic Effect of Chlamydia Trachomatis

CT, like other intracellular pathogens, can cause alterations in gene expression and protein production. These changes can induce host genome duplication leading to aneuploidy and chromosome instability [3,11]. These effects on the host cell during CT infection are possible contributing factors to the epidemiological association between CT and CC with or without previously HPV-infected patients, though a direct molecular mechanism has yet to be found [3,11,15].

**Figure 3:**
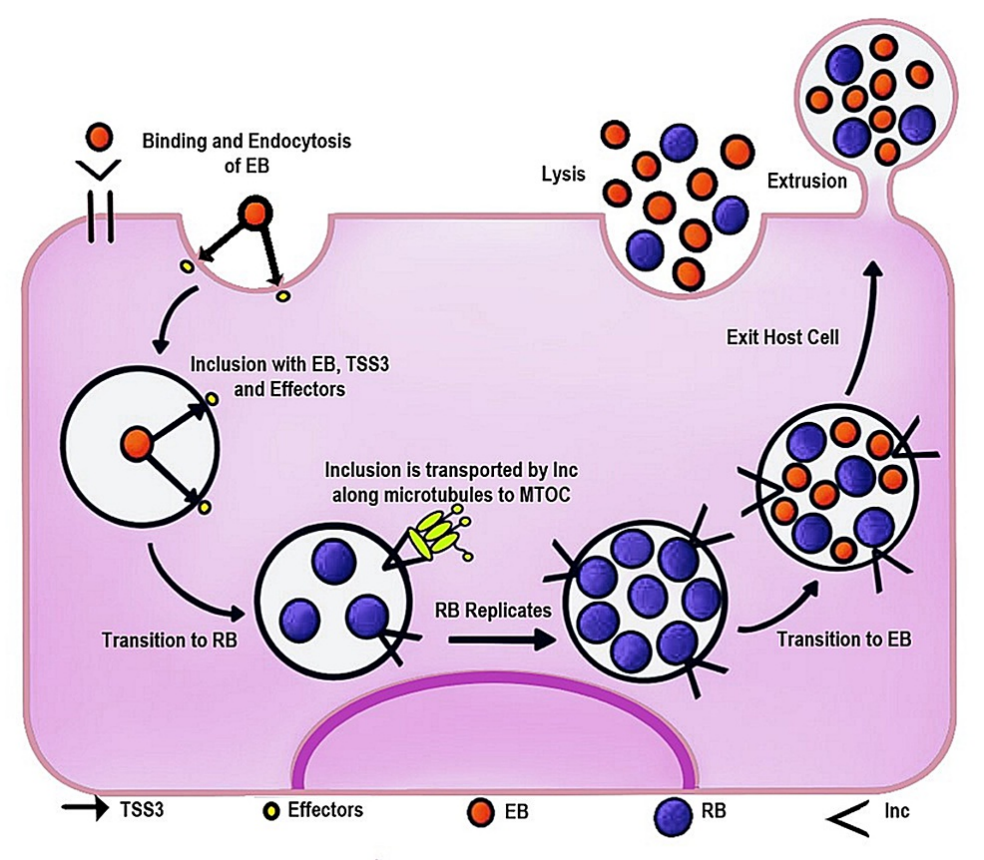
Life cycle of Chlamydia trachomatis EB: elementary body; RB: reticulate body; TSS3: type III secretion system; Inc: inclusion membrane proteins; MTOC: microtubule-organizing center [23]

CT goes through a biphasic life cycle including an elementary body (EB), which enters the host cell by inducing endocytosis, and reticulate bodies (RB), which are differentiated and metabolically active EBs [22]. While in the cell, CT remains within a vacuole surrounded by a membrane, called an inclusion, in which it replicates [15,21]. Figure 3 illustrates CT's life cycle from its invasion to its exit from the host cell. We can appreciate its transition from EB to RB and back to EB, the form in which it exits the cell via extrusion or cell lysis ready to infect other cells. 

These inclusions are closely associated with the host cell’s centrosomes; dependent on the motor protein dynein, they migrate along the microtubule-organizing center (MTOC), resulting in defects in centrosome formation and localization. It has been reported that this interaction between CT vacuoles and the MTOC generates the formation of supernumerary centrosomes, giving rise to multipolar mitotic spindles and hindering accurate chromosome segregation [3]. Others argue that it is CT-induced cytokinesis failure that leads to multinucleation [11].

As an obligate intracellular pathogen, CT is required to alter many aspects of the host cell’s functions to make it hospitable to survive. It has multiple secretion systems to efficiently deliver effectors with cytopathic effects including degradation of host proteins, centrosome amplification, and multinucleation of host cells [11]. It has also been reported that CT triggers activation of oncogenic pathway components Ras-Raf-MEK-ERK and produces reactive oxygen species (ROS) to support its growth [22]. Supernumerary centrosomes are seen in carcinomas of many origins, as the addition or subtraction of chromosomes due to mitotic defects can significantly increase tumor progression [3].

Knowlton et al. in 2011 demonstrated that CT causes mitotic spindles defects independently of its effects on centrosome amplification by inhibiting the spindle assembly checkpoint (SAC) delay. The SAC is a monitoring mechanism that regulates the attachment of microtubules to their corresponding kinetochore, delaying the onset of anaphase until the microtubules are organized with their corresponding chromosome [3]. Their data suggest that CT inhibits the cell’s ability to suppress defects on mitotic spindle organization, and causes the host cell to prematurely exit mitosis without corrections [3,15].

Another study describes a mechanism by which CT induces a cleavage furrow defect that results in the creation of multinucleated cells, a phenotype well-associated with tumorigenesis. After CT infection, host-signaling proteins involved in the initiation and stabilization of cleavage furrows are displaced, leading to the formation of asymmetrical, unilateral cleavage furrows. According to them, this is caused by the mere physical presence of CT at the cell equator. The study’s live-cell imaging indicated partially formed or regressed furrow at the inclusion’s side, suggesting that the initiation of the cleavage furrow was impaired. The large inclusion or vacuole also positioned the mitotic spindles eccentrically. The study suggests that all these defects were the result of physical displacement instead of degradation of host-signaling proteins [15].

However, Brown et al. demonstrated that multinucleation is due in its entirety to cytokinesis failure. By using live-cell imaging, they were able to examine infected dividing cells directly. They observed that 80.5% of infected cells became multinucleated many hours after mitosis was completed. This transformation could only be justified by two possible mechanisms: fusion of closely associated daughter cells after the completion of cytokinesis or failure in late cytokinesis after diffusion is blocked by the formation of the midbody. They found that the cleavage furrows appeared undisturbed by the CT inclusions and that failure occurs at a later step than the formation and function of the contractile rings, most likely during abscission. After ruling out fusion, they determined at what point cytokinesis fails. They observed fully formed midbodies in mitotic Chlamydia-infected cells, indicating that these cells successfully progressed through the actin-myosin contractile ring stage into this late cytokinesis stage. As previously stated, infected cells exit mitosis prematurely before metaphase is complete. This is associated with chromosome segregation defects such as chromosome non-disjunction and lagging chromosomes. Brown et al. observed that the infected cell population had a significant increase in the number of cells with lagging DNA in the midbody between daughter cells. These data further suggest that chromosome segregation defects and inhibition of SAC directly contribute to CT-induced cytokinesis failure [11].

In 2013, Knowlton et al. demonstrated that Chlamydial infection transforms 3T3 cells in soft agar, resulting in anchorage independence and increased colony formation. Also, for the first time, they observed how Chlamydia infects cells in vivo by infecting mice with Chlamydia muridarum, which resulted in increased cervical cell proliferation and evidence of dysplasia. They showed that for Chlamydia to induce cell defects such as centrosome amplification, multipolar spindles, and multinucleation, host cell division, and not the co-expression of HPV oncogenes E6 or E7, is the primary requirement and the likely defining factor for CC development [10].

CT is also associated with alterations to host histones, in particular the sustained upregulation of PH2AX and H3K9me3, both hallmarks of DNA double-stranded breaks (DSBs) and senescence-associated heterochromatin foci (SAHF) respectively. ROS induced by CT contributes to the continuation of DSBs, which in turn elicit SAHF formation in an ERK-dependent manner. In addition, CT inhibits recruitment of the DNA damage response (DDR) proteins pATM and 53BP1 to sites in need. Nonetheless, even with impaired DDR, the cells continue to proliferate supported by oncogenic signals involving ERK, Cyclin E, and SAHF. Thus, CT infection causes DSB generation via the uncommon combination of impaired repair and pro-survival signaling, which could predispose host cells to genomic instability and transformation [22].

Most recently, global phosphoproteomics and transcriptomic analysis have revealed CT and CT-regulated host phosphoproteins. These proteins are predominantly related to transcription regulation, cellular growth, proliferation, and cytoskeleton organization, thereby uncovering the remarkable impact CT has on host-cell signaling and cell behavior. Bioinformatic analysis revealed that the phosphosites upregulated and downregulated during infections, and regulate key processes that can contribute to the diverse hallmarks of cancer, such as cellular proliferation. The phosphorylation status of regulated transcription factors has been identified and they are also found to be a substrate for ERK/MAPK. Functional analysis of this data confirmed the involvement of the phosphoproteins in epithelial-to-mesenchymal transition (EMT) along with the essential role of ERK 1/2, ETS1, and ERF for CT replication. This study has revealed the extent to which CT induces signaling and provides insight into its potential as a procarcinogen [12].

Chlamydia Trachomatis and Cervical Cancer: An Epidemiological Association

Several studies have suggested that HPV and CT coinfection are associated with the development of CC. CT infection causes disruption of the epithelial tissue facilitating HPV persistence and progression to cervical malignancy [3]. This could also be a product of shared common transmission routes and risk factors [8]. Data has revealed up to a four-fold higher risk of hrHPV infection in CT-positive women compared to the negative group, and nearly a two-fold duration of the hrHPV infection [24]. Nonetheless, CT and HPV coinfection's effects and clinical consequences are poorly explored [8]. Table 2 presents selected studies that met our criteria supporting the hypothesis that CT infection is associated with HPV and/or cervical changes.

**Table 2:** Positive correlation between CT, HPV, and cervical cancer CT: Chlamydia trachomatis; HPV: human papillomavirus; HrHPV: high-risk human papillomavirus; LSIL: low-grade squamous intraepithelial lesion; CC: cervical cancer

Author	Year of publication	Study design	Population characteristics	Sample size	Outcome
Chen et al. [8]	2020	Cross-sectional study	Gynecology clinic in southern Hunan, China	5,006	CT infection was associated with HrHPV infection with an odds ratio of 1.74 (95% CI: 1.10–2.74, p=0.017)
Ssedyabane et al. [4]	2019	Cross-sectional study	Age: 25-80 years; hospital in southwestern Uganda	93	There is a likelihood of association between HPV-CT coinfection and the cytological diagnosis of LSIL (Spearman's rho=0.2784, prob >|t|=0.0069)
Madaan et al. [7]	2019	Cross-sectional study	Age: 18-45 years; STI clinic in New Delhi, India	90	A highly significant association was found between HPV-CT coinfection and cervical abnormal cytology (p=0.001)
Lv et al. [24]	2019	Cross-sectional study	Age: 20-70 years; outpatient clinic in Shanghai, China	826	Data evaluated showed that CT (OR: 3.538) is a risk factor for hrHPV infection (p<0.05)
Mancini et al. [25]	2018	Cohort study	Multicenter, Italy	164	A high percentage (15/16; 94%) of CT-HPV coinfections have high-grade cervical lesions more frequently than those infected with HPV only
Zhu et al. [6]	2016	Meta-analysis	22 studies	4,291	CT was significantly linked to increased CC risk in prospective studies (OR: 2.21, 95% CI: 1.88-2.61)
Arnheim Dahlström et al. [26]	2011	Prospective cohort study	4 major biobanks in Nordic countries	1,000,000	Previous exposure to CT; had a strongly increased risk for CC (OR: 1.9; 95% CI: 1.5-2.3)

Other studies have found no difference between hrHPV infection with negative or abnormal cervical cytology and CT coinfection [3]. Even after adjusting for demographic factors, including education, marital status, and smoking, Robial et al. did not find an association with CT infection [27]. Table 3 presents selected studies that met our criteria of refuting the hypothesis that CT infection is associated with HPV and/or cervical changes.

**Table 3:** Negative correlation between CT, HPV, and cervical cancer HPV: human papillomavirus; CT: Chlamydia trachomatis; HrHPV: high-risk human papillomavirus

Author	Year of publication	Study design	Population characteristics	Sample size	Outcome
Abu-Lubad et al. [28]	2020	Case-control study	Age: 20-80 years; multicenter, Jordan	144	A lack of coinfection was observed between HPV and CT in both cancer types
Sangpichai et al. [3]	2019	Cross-sectional study	Khon Kaen University, Thailand	150	CT infection was not significantly associated with hrHPV and abnormal cytology
Robial et al. [27]	2017	Cross-sectional study	Age: 18-64 years; cancer-screening project, Sao Paulo, Brazil	1,481	No association was found between abnormal cervical cytology and positive CT [OR: 1.21 (0.46-3.2)]
Smelov et al. [29]	2016	Case-control study	Age:16-89 years; Sweden	1,553	CT was not associated with increased risks of invasive adenocarcinoma or its precursor, adenocarcinoma in situ
Bhatla et al. [5]	2013	Cross-sectional study	Age:30-74 years; hospital in New Delhi, India	600	Subjects with positive hrHPV and CT showed no significant association with abnormal Pap smears, compared with hrHPV infection alone [p=0.210, OR: 0.3 (0.0-2.5)], or histopathology CIN2 or greater [p=0.341, OR: 0.342 (0.034-3.424)]
Calil et al. [30]	2011	Cross-sectional study	Primary care units in southern Brazil	86	The presence of CT infection does not seem to be associated with cervical carcinogenesis

Limitations

Our review has some limitations. Firstly, the research was only conducted through PubMed and Google Scholar databases, and only free articles and abstracts were selected; therefore, we might not have covered all findings of the relationship between CT and CC. Additionally, this review engaged with different study designs, populations, and sample sizes in a relatively short period of 10 years (2011-2021). The role of CT in the development of CC is still controversial and most evidence is epidemiological and needs further study. 

## Conclusions

HrHPV has been shown to be a necessary but not sufficient cause of CC. Only a small number of exposed women will have a persistent infection that will eventually progress to cancer. Prior studies have evaluated the role of other microorganisms that cause chronic inflammation as potential risk factors for the transmission and persistence of HPV, thereby promoting progression to cervical neoplastic changes. The most common sexually transmitted bacterial infection is caused by CT, and it has been epidemiologically associated with increasing the risk of CC, with or without HPV coinfection. Globally, the methodology used to collect data on this relationship is observational studies and meta-analyses. Researchers have been searching for different ways to approach the investigation of events that occur during CT infection and how that could possibly enhance tumorigenesis. It has been noted that CT causes significant changes to gene expression and protein production at the transcriptional, translational, and post-translational levels. It suppresses the cell’s ability to correct defects and causes it to exit mitosis prematurely. CT affects cervical cells via the uncommon combination of impaired repair and pro-survival signaling.

We hypothesize that induction to cervical metaplasia is what creates target cells for HPV coinfection and persistence, but despite all the research, the role of CT in CC is not entirely understood. Some studies have found a correlation between CT and HPV coinfection with cervical changes, while others have found no significance at all. Since CT can be treated if diagnosed, and a large percentage of women are asymptomatic, measures should be taken to promote yearly screening tests worldwide. At the moment, we are still lacking a disease model that could clarify the role of CT. A multicenter, large-sample, case-control study will be needed in the future to address these inconsistencies.
