# Correction: The Role of Chlamydia Trachomatis in the Pathogenesis of Cervical Cancer

**DOI:** 10.7759/cureus.c57

**Published:** 2022-03-03

**Authors:** Ana P Arcia Franchini, Beshoy Iskander, Fatima Anwer, Federico Oliveri, Kakargias Fotios, Priyanka Panday, Pousette Hamid

**Affiliations:** 1 Research, California Institute of Behavioral Neurosciences & Psychology, Fairfield, USA; 2 Internal Medicine, Bon Secours Mercy Health - St. Elizabeth Youngstown Hospital (NEOMED), Ohio, USA

Two errors were identified by a reader after publication and immediately brought to the attention of the authors. After a thorough review of the published article, the following errors have been identified and corrected:

Chlamydia was incorrectly referred to as a gram-positive organism:

- Introduction and Background, fourth paragraph, first sentence: "Gram-positive" corrected to "**Gram-negative**"

Chlamydia was also incorrectly referred to as the most common sexually transmitted infection:

- Introduction and Background, third paragraph, second and third sentences: "It is estimated that there are 357 million new infections due to one of the four most common STIs each year, including Chlamydia, gonorrhea, syphilis, and trichomoniasis [4]. Among the previously mentioned STIs, Chlamydia is the most common STI, representing 20-40% of all STIs worldwide, and with more than 1.4 million infections in the United States alone [3]." corrected to "It is estimated that there are 357 million new infections due to** one of these four common STIs** each year: Chlamydia, gonorrhea, syphilis, and trichomoniasis **[1]**. Among the previously mentioned, Chlamydia is the **most common bacterial STI, representing 20-40% of all STIs with more than 1.4 million infections in the United States alone [7]."**

Cureus and the authors deeply regret that these errors were not caught prior to publication.

